# Electroretinographic features of the retinopathy, globe enlarged (*rge*) chick phenotype

**Published:** 2007-04-04

**Authors:** Fabiano Montiani-Ferreira, Gillian C. Shaw, Andrew M. Geller, Simon M. Petersen-Jones

**Affiliations:** 1 Department of Small Animal Clinical Sciences, College of Veterinary Medicine, Michigan State University, East Lansing, MI; 2 U.S. Environmental Protection Agency, Research Triangle Park, NC

## Abstract

**Purpose:**

The purpose of the study was to characterize the electroretinographic features of the autosomal recessive retinopathy, globe enlarged (*rge*) phenotype, in chickens (*Gallus gallus*).

**Methods:**

Dark-adapted, light-adapted intensity series and light-adapted 30 Hz flicker responses were recorded from *rge* and age matched normal control chicks from one to 270 days of age. Retinal sections from *rge* and control retinas were examined in 7 and 270-day-old chicks.

**Results:**

Electroretinogram (ERG) thresholds of *rge* birds were raised, the intensity response plots were shifted toward brighter intensities, and retinal sensitivity was reduced. The leading slope of the dark- and light-adapted a-waves was more shallow than normal, suggesting altered photoreceptor responses. The inner retinal components to the ERG were also abnormal; there was a marked lack of oscillatory potentials and an abnormally smooth and broad shape to the b-wave. Additionally, the b-wave was supernormal in response to brighter stimuli in the earlier stages of the disease. There was a progressive deterioration in ERG amplitudes with age that mirrored a slowly progressive thinning of the photoreceptor layer.

**Conclusions:**

The *rge* chicken has unusual ERG changes from an early age with altered waveforms and initially they develop a supernormal b-wave. This is followed by a progressive reduction of ERG amplitudes with age. The changes suggest that both photoreceptor and inner retinal responses are abnormal. Additional studies are needed to further elucidate the origin of the abnormal ERG components in the *rge* chick.

## Introduction

The chick retina has some significant differences from the mammalian retina. It has four types of single cone with peak sensitivity to ultraviolet light as well as, short, medium, and long wavelength light (UV, S, M, and L cones, respectively) [1,2]. The cones contain different colored oil droplets that act to shift the peak sensitivity of a cone to a wavelength longer than that of maximum absorbance of the photopigment. The droplets also narrow the spectral sensitivity (For a review, see [3]). As is the case with other birds, chicken retinas also contain double cones that consist of principle and accessory components with peak light absorbance between that of M and S cones. It has been suggested that the double cones play a role in luminance-based tasks, such as motion detection, and do not play a major role in color recognition [1]. Chicks have a single form of rod photoreceptor with peak absorbance similar to that of the M cone. The chicken retina is cone dominated. Morris reported that the central retina had 14% rods, 32% double cones, and 54% single cones while the peripheral retina had 33% rods, 30% double cones, and 37% single cones [4]. The ratio of single cone types L: M: S + UV is 5: 2: 1 [4]. Compared to commonly studied mammalian species, such as mice, rats, dogs and humans where the cones account for 5% or less of the total photoreceptors, the higher proportion of cones in the chicken is reflected in the electroretinogram (ERG) of the chick. The chick ERG is dominated by the cone responses with the rod component being of relatively low amplitude in comparison to the commonly studied mammalian species. Additionally, it has been reported there is a strong circadian effect on chick rod function, whereby rod ERG responses are greater at night than during the day [5].

The chick is commonly used for studies of ametropia, and the globe readily undergoes differential growth when the retinal image is occluded or defocused or when the chick is raised under constant light exposure (See [6] for a review). The chick is also emerging as a model of retinal dystrophies with a number of spontaneously occurring disorders being recognized and studied. The chicken retinal dystrophy described in great detail is that of the retinal degeneration (*rd*) chicken. The *rd* chicken has an autosomal-recessive condition that causes homozygous-affected birds to be blind at hatch and to lack ERG responses [7]. At hatch the retinal morphology appears normal, but there is a progressive photoreceptor degeneration. The *rd* chicken is a model for Leber Congenital Amaurosis type 1 in that it is, caused by a null mutation in the retinal guanylate cyclase gene [8]. Other retinal dystrophies described in chickens include the retinal dysplasia and degeneration (*rdd*) chick, which has an extinguished ERG by three weeks of age. The condition is sex-linked, although the causal mutation has not been reported [9]. The delayed amelanotic (*dam*) chicken has a phenotype that involves cutaneous and ocular amelanosis. The retinal pigment epithelial (RPE) of *dam* chickens lacks phagocytic activity, and a retinal degeneration develops, presumably secondary to RPE dysfunction. The main ERG changes observed in *dam* chickens are a generalized decrease in waveform amplitudes, which parallel the retinal degenerative changes. C-waves are affected more than a-waves, and a-waves more than b-waves. Dark-adapted responses are affected more than light-adapted responses [10].

We have previously described the clinical and histological features of a more recently identified chicken retinal dystrophy known as retinopathy, globe enlargement (*rge*). This is a naturally-occurring autosomal recessive retinal disorder leading to blindness [11,12]. The gene locus has been mapped to chicken chromosome one [13]. Affected birds lose functional vision over the first few weeks of life as assessed by optokinetic responses, as we have previously reported, and then develop a progressive globe enlargement [14]. We have previously described the histological features of the condition in some detail [15].

Briefly, *rge* chickens have a slow reduction in retinal photoreceptors with age, part of which might be accounted for by the increase in globe size that develops secondarily in this model. However, before they develop an increase in globe size that might result in retinal changes, the *rge* chicks show abnormalities in the photoreceptors and outer nuclear layer. In 7- day-old *rge* chicks, the photoreceptor cell bodies appear slightly dilated, and synaptic terminals in the outer plexiform layer are disorganized compared to the regular two-layered arrangement seen in normal chicks. Smooth endoplasmic reticulum in the accessory cones of mutant chicks is present internal to the outer limiting membrane, while it is present in the inner segments of control chicks as part of the hyperboloid. Glycogen deposits are displaced from the normal position in the inner segment toward the cytoplasmic perinuclear areas of the accessory cells of double cones and of rod cells. These misplaced deposits increase in size with age.

On ultrastructural examination, abnormalities in photoreceptor pedicles and spherules are detectable by seven days of age. The pedicles and spherules are increased in size compared to controls, not so electron dense, and often contain sets of numerous flattened (tubuliform) small vesicles and multivesicular bodies. Immunohistochemistry reveals that opsin mislocalization occurs in rods by 13 days of age and progresses as the birds age. Mislocalization of opsin is a common finding in a retina undergoing degenerative changes. This finding has been described in several retinal degeneration models, including those resulting from both hereditary and environmental disease, and has been shown by in vitro studies to be able to trigger apoptotic death of rods [16].

Our preliminary ERG studies suggested that in contrast to many retinal dystrophies where the loss of vision results from photoreceptor dysfunction or degeneration, which can be detected by loss of ERG responses, the rapid loss of vision occurring over the first few weeks of age in the *rge* chick is not mirrored by a corresponding diminution of ERG tracings [14]. Instead, the ERG tracings have a characteristic abnormal waveform and only a slow reduction of amplitudes with age.

The present study was performed to characterize these apparently unusual ERG abnormalities of the *rge* chicken in more detail.

## Methods

### Animals

A breeding flock of rge chicks was maintained at the vivarium of the College of Veterinary Medicine, Michigan State University under 12 h:12 h light-dark cycles. All procedures were conducted in accordance with the US Public Health Service (Public Health Service Policy on Humane Care and Use of Laboratory Animals) and approved by the Michigan State University All-University Committee on Animal Use and Care.

### Electroretinographic recording

Dark-adapted and light-adapted white light intensity series ERGs were recorded from affected birds and normal control birds at ages ranging from 1 to 270 days. The left eye was used for ERG recording. The pupil was dilated with 1% vecuronium bromide (ESI Lederle, Philadelphia, PA). Anesthesia was induced and maintained with isofluorane. A pulse-oximeter (Vet/Ox 4400, Heska Inc., Fort Collins, CO) was used to monitor heart rate and oxygen saturation for the duration of the recording session. Body temperature was maintained using a heating pad.

Conjunctival stay sutures of 4-0 silk (Ethicon, Inc., Cornelia, GA) were used to position the eye in primary gaze. Under a dim red light, a Burian-Allen bipolar corneal contact lens electrode (Hansen Labs, Coralville, IA) was lubricated with hydroxypropyl methylcellylose (Goniosol, Alcon Inc., Fort Worth, TX) and placed on the cornea. A ground electrode was placed subcutaneously in the hind limb.

Full-field (Ganzfeld) flash intensity-series ERGs were recorded with a UTAS-E 3000 Electrophysiology unit (LKC Technologies Inc, Gaithersburg, MD) with the filter bandpass set between 1 and 500 Hz.

### Dark-adapted intensity series electroretinography

A preliminary study showed little difference in response thresholds or ERG waveforms between birds dark-adapted for 20 min compared to those dark-adapted for 45 min. Therefore, to keep anesthesia times short, we selected 20 min of dark adaptation. ERGs, in response to white light flashes, were recorded at 12 intensities ranging from -2.4 to 2.8 log candela-seconds (cdS)/m^2^, (Flash intensity was calibrated with a Research Radiometer IL 1700 with SED033) silicon light detector, (International Light, Inc. Newburyport, MA). For intensities between -2.4 to -0.79 log cdS/m^2^, ten flashes were averaged and from -0.39 to 2.8 log cdS/m^2^ three flashes were averaged. Inter-stimulus intervals were increased from 1 s at low intensities to 3 min at higher intensities to avoid light-adapting the rods. In selected instances following the recording of the brightest flashes some dim-light flashes were also rerecorded and compared with the original tracings at that intensity to ensure that the eyes had remained dark adapted during the procedure.

### Light-adapted intensity series electroretinography

Following the dark-adapted intensity series the birds were light adapted to a white background light of 30 cd/m^2^, for 10 min. The same intensities used for the dark-adapted ERGs imposed on the background light were repeated to record the light-adapted responses.

A white flash stimuli of 0.4 cdS/m^2^ superimposed on a background light of 30 cd/m^2^ was used to record 30 Hz flicker ERG.

### Data analysis

The a- and b-wave amplitude and implicit time were measured for each averaged ERG response. The a-wave amplitude was measured from the onset of stimulus to the trough of the a-wave, b-wave amplitude from the trough of the a-wave (or from baseline in the absence of an a-wave) to the peak of the b-wave. A-wave implicit time was the time measured from the onset of the stimulus to the time when the maximal a-wave trough occurred and b-wave implicit time from the onset of the stimulus to the time when the peak b-wave was present.

A criterion threshold value of 5 μV was selected for a- and b-wave responses. This was derived for each bird by plotting the a- and b-wave intensity response curves.

Repeated measures ANOVA was performed to determine the statistical significance of the differences in ERG parameters between affected and control birds at a given fixed age and intensity and between ages for the same genotype at a fixed intensity. If any statistically significant difference was found the data were further analyzed (P values adjusted) using post hoc comparisons with Fisher's or Tukey-Kramer tests. Data were deemed significant when p<0.05. This analysis was performed using statistics computer software (SAS 2001-version 8.2, SAS Institute Inc., Cary, NC).

Analysis of a-wave parameters included comparisons of a-wave threshold, maximal a-wave amplitude recorded from the intensity series, a-wave implicit time, Naka-Ruston analysis of a-wave intensity response curves (see next section), and a comparison of the slope of the a-wave by comparison of normalized intensity matched a-waves.

B-wave analyses compared dark-adapted and light-adapted b-wave thresholds, maximal b-wave amplitudes, b-wave implicit times, and Naka-Ruston fitting.

### Naka-Ruston fitting

To further quantify differences in a- and b-wave intensity response curves these were fit with a 3-parameter Hill equation, also known as a Naka-Rushton function in vision science. This function is as follows: V(I)=(Vmax x In)/(In+kn), where V denotes the amplitude of the a- or b-wave for a given flash intensity I, Vmax is the upper asymptote of the amplitude versus intensity function, k, the flash intensity yielding a response amplitude of 1/2Vmax, and n, an exponent affecting slope. In some cases, n is restricted to = 1; n was allowed to vary freely in these curve-fits. k is often considered to reflect sensitivity, since it represents a constant criterion (1/2Vmax) that determines where the amplitude versus intensity function is located along the flash intensity axis. The curves were fit using SigmaPlot 2001, version 7.101 (SPSS, Inc), which employs a Marquardt-Levenberg algorithm to perform least-squares fits.

For the 30 Hz flicker responses, amplitude (trough to peak) and implicit times (flash onset to peak amplitude) were measured.

### Retinal histology

Four 7 day old and four 270 day old chicks were euthanized using a CO_2_ chamber. Afterwards, bilateral enucleation was performed. The eyes were hemi-sected at the equator, the vitreous body removed, and the posterior segment of the eye immersed into 3% paraformaldehyde, and 2% glutaraldehyde, in phosphate-buffered saline (0.1 M, pH 7.3), for 3 h at room temperature. A square-shaped tissue sample (about 2x2 mm) per eye was collected from the central retina, subsequently post-fixed in 1% OsO4 at 4 °C for 2 h, washed in distilled water, then dehydrated in acetone and finally embedded in an Araldite-based resin (Durcupan, Fluka, Seelze, Germany). Semi-thin (0.5-1 μm) sections were cut using a Reichert-Jung Ultracut ultra-microtome (Reichert-Jung, Wien, Austria), using a glass knife. Semi-thin sections were stained with toluidine blue solution and examined by light microscopy and images recorded with a Polaroid DMC digital camera (Polaroid, Waltham, MA) mounted on a Nikon Eclipse E400 Microscope (Nikon, Melville, NY).

## Results

### Raw waveforms and response thresholds

**Figure 1:**
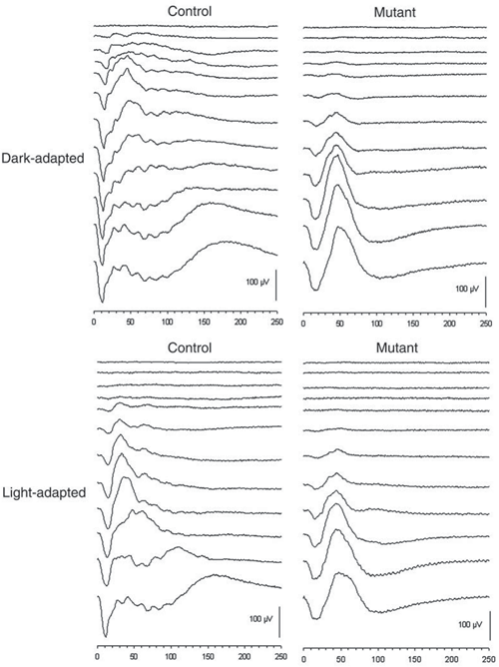
Representative dark-adapted and light-adapted electroretinogram recordings from a control chick and an *rge* (mutant) chick aged seven days. The *rge* chick had an elevated response threshold for both dark-adapted and light-adapted waveforms. The shape of the *rge* electroretinogram waveform was quite different from that taken of the control chick. The a-wave slope is less steep than that of the normal chick and the a-wave implicit time increased. The b-wave is smoother due to the lack of oscillatory potentials and is greater in amplitude than that of the control bird at the brighter flash intensities. Flash intensities from top to bottom were as follows: -2.4, -2.0, -1.42, -1.19, -0.79, -0.39, 0.00, 0.39, 0.85, 1.4, 2.3, and 2.9 log cdS/m^2^. Vertical bar represents 100 μV; X-axis is time in mSec.

**Figure 2:**
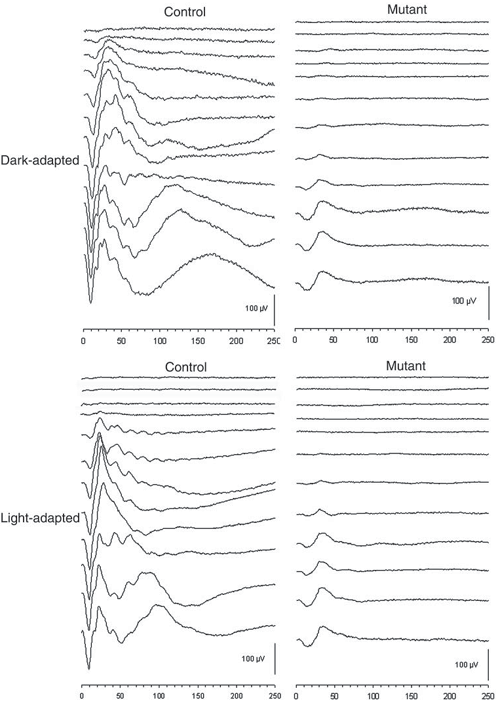
Representative dark-adapted and light-adapted electroretinogram recordings from a control chick and an *rge* (mutant) chicken aged 270 days. The control bird ERG intensity series are typical for an adult chicken and show greater amplitudes than those recorded from the 7-day-old control bird. The 270-day-old control bird's dark-adapted and light-adapted thresholds were lower than that seen in the 7-day-old control chick (Compare with Figure 1). The dark-adapted and light-adapted ERG thresholds of the *rge* bird were greater than that of the control and greater than that of the 7-day-old *rge* chick (See Figure 1). The shape of the ERG waveform of the 270-day-old *rge* bird was similar to that of the 7-day-old *rge* chick, although the amplitudes were lower, the implicit times increased, and the b-wave was no longer supernormal. The light-adapted and dark-adapted responses of the 270-day-old *rge* bird were similar. Flash intensities from top to bottom were as follows: -2.4, -2.0, -1.42, -1.19, -0.79, -0.39, 0.00, 0.39, 0.85, 1.4, 2.3, 2.9 log cdS/m^2^. Vertical bar represents 100 μV; X-axis is time in mSec.

**Figure 3:**
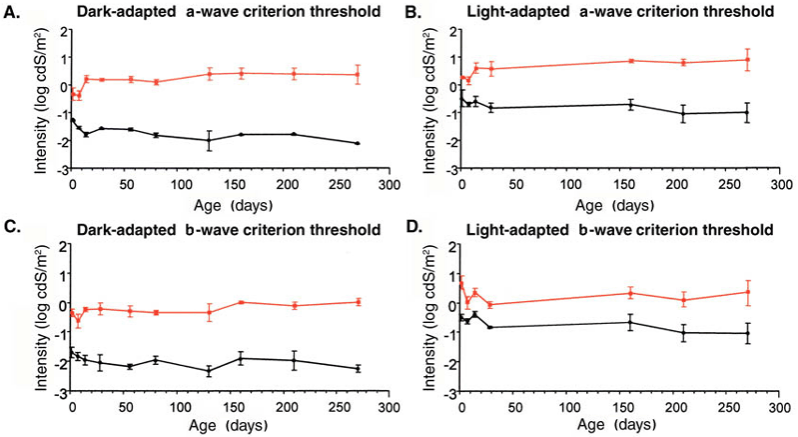
Mean flash intensity required to elicit a 5 μV criterion response threshold response (±1 SEM) plotted against age. **A** and **B** show a-wave thresholds for dark-adapted and light-adapted birds, respectively. **C** and **D** present b-wave thresholds for dark-adapted and light-adapted birds, respectively. Black lines represent control birds, and red lines represents *rge* birds. In the control birds the response thresholds decreased slightly over the first few weeks of life then plateaued. The thresholds of the *rge* birds were elevated, but they also decreased slightly from one to seven days of age, increased at 14 days of age, then remained similar over the time period of the study. n is between three and seven birds at each age.

Dark- and light-adapted intensity series ERG waveforms from *rge* chicks and age-matched controls are shown in Figure 1 (at seven days of age) and Figure 2 (at 270 days of age). As these figures show, the *rge* chicks had an increased response threshold for both dark- and light-adapted responses. To further investigate the response thresholds, we plotted mean 5 μVolt criterion thresholds for both a- and b-waves (Figure 3). The mean criterion a-wave and b-wave thresholds of *rge* chicks were significantly elevated at all ages with the exception of the light-adapted a-wave threshold at one day of age. Dark- and light-adapted mean 5 μV criterion response thresholds (a- and b-wave) decreased in control birds as retinal function matured over the first few weeks of life. The mean response thresholds of the *rge* chicks also decreased over the first week of age but then rose one week later and remained fairly constant for the duration of the study. The dark- and light-adapted thresholds of the *rge* birds were similar, whereas in the controls both a- and b-wave dark-adapted mean criterion thresholds were at least one log unit lower than the light-adapted thresholds. This feature can be seen in the intensity series in Figure 1 and Figure 2, and it is shown for the mean criterion threshold in Figure 3.

### Alteration in waveform shape

**Figure 4:**
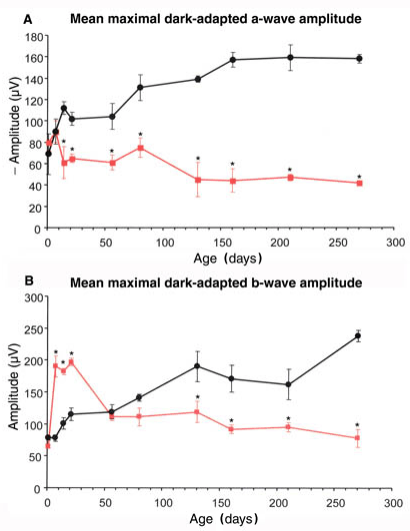
Mean maximal dark-adapted a- and b-wave plotted against age. Mean (1x±SEM) dark-adapted maximal a-wave (**A**) and b-wave (**B**) recorded from *rge* (red lines) and control birds (black lines). **A**: After an initial increase in maximal a-wave between one and seven days of age, the a-wave amplitude in the *rge* group decreased by 14 days of age. It remained fairly constant for the rest of the ages investigated, whereas that of control birds increased further with age. **B**: The *rge* birds had a supernormal maximal b-wave from 7 to 28 days of age followed by a decrease in amplitude with increasing age. n=6 control and five *rge* birds; asterisk (*) indicates the amplitude of the *rge* birds was significantly different from the control birds. A p<0.05 was considered significant.

**Figure 5:**
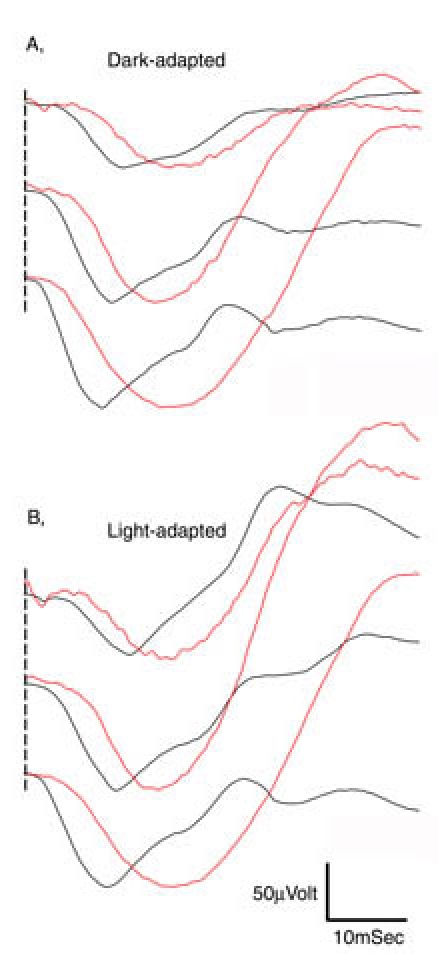
Comparison of normalized a-waves from dark-adapted and light-adapted *rge* and control birds. Comparison of normalized a-wave response from dark-adapted (**A**) and light-adapted (**B**) *rge* (red tracings) and control (black tracings) birds. The waveforms represent the averaged waveforms from eight *rge* and eight control birds aged 13-22 days old. For each comparison the averaged waveforms from the *rge* birds were normalized to the peak amplitude of the mean control bird a-wave. Flash intensities from top were 0.39, 1.36, and 2.39 log cdS/m^2^. Horizontal bar is 10 mSec. Vertical bar of 50 μV refers only to the control waveforms, since the waveforms of the mutant birds were normalized. Initiation of the a-wave down slope was delayed, the initial portion of the leading slope was less steep, and the implicit time was increased in the *rge* birds compared to the controls.

In addition to the increased response thresholds, the ERG of the *rge* chick was abnormal in shape (Figure 1). This was apparent from one day of age (waveforms not shown). With age the amplitudes of the *rge* chick ERG waveforms decreased (compare Figure 2 with Figure 1 also see Figure 4) but the shape of the waveform remained similar. The ERG waveform of *rge* chicks had a less steep a-wave leading edge (also see Figure 5), a wider trough, a more slowly rising initial phase of the b-wave, and a lack of oscillatory potentials (OPs) imposed on the b-wave compared with the ERG of the controls. The most striking feature of the intensity series from the seven-day-old *rge* chick was the development of an abnormally large amplitude b-wave (supernormal b-wave) in response to the brightest flashes in both the dark- and light-adapted state. This supernormal b-wave amplitude developed during the first week after hatch and peaked at three to four weeks of age after which the peak amplitude decreased with age. By 49 days of age, it was similar to that of controls (Figure 4).

### Intensity response plots

**Figure 6:**
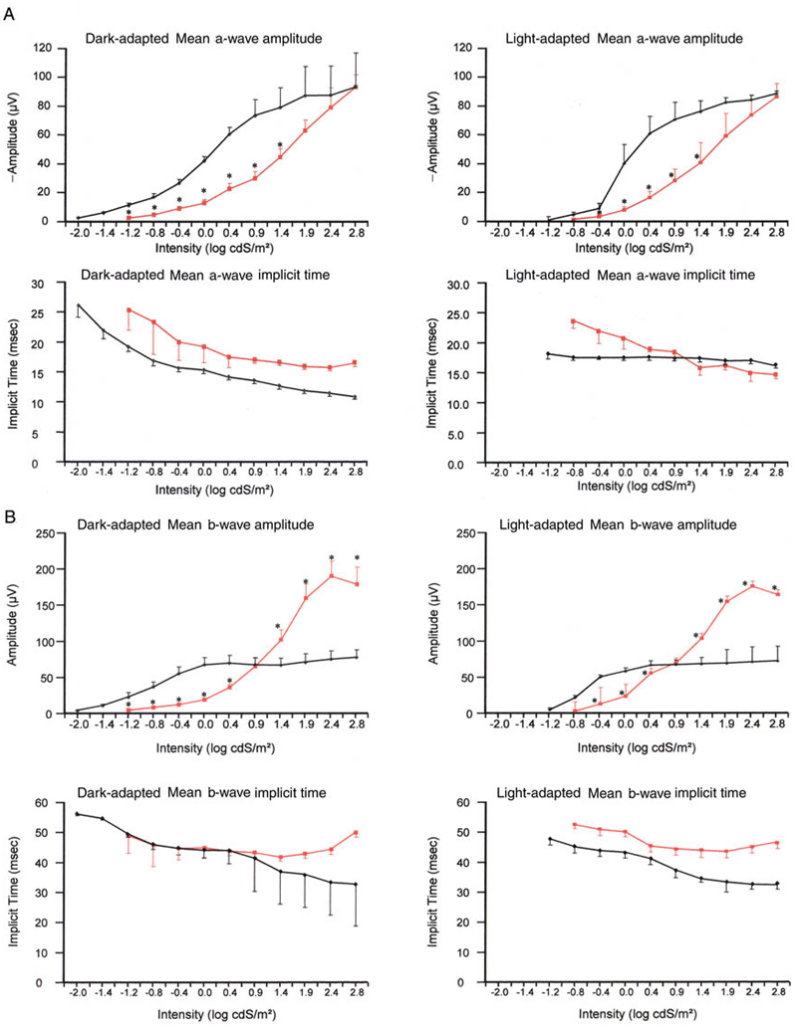
Mean dark- and light-adapted a- and b-wave amplitude and implicit times plotted against flash intensity for 7-day-old *rge* and control chicks. **A**: Mean (±SEM) dark-adapted and light-adapted a-wave amplitude and implicit times plotted against flash intensity for 7-day-old *rge* chicks (red lines) and control chicks (black lines). **B**: Mean (±SEM) dark-adapted and light-adapted b-wave amplitude and implicit time plotted against flash intensity for 7-day-old *rge* chicks (red lines) and control chicks (black lines). N=7 control and seven *rge* birds. Asterisk (*) indicates that the amplitude of the *rge* birds was significantly different from the control birds. A p<0.05 was considered significant.

To further investigate the amplitude and timing of the a- and b-wave, we plotted the amplitude and implicit time against flash intensity (Figure 6 shows the results at seven days of age). The dark- and light-adapted a-wave amplitude intensity-response (I:R) plot (Figure 6A upper graphs) showed that the a-wave of the *rge* chicks at seven days of age was significantly lower than that of the controls for the lower intensities of stimulus. When the flash intensity was increased, the control chick a-wave tended toward saturation. However, the *rge* chick mean a-wave continued to increase with increasing flash intensity and was similar in amplitude to the mean a-wave of the control birds at the brightest flash intensity (2.8 log cdS/m^2^, see Figure 6A). The dark-adapted a-wave implicit time of the control chicks decreased with increasing flash intensity, while the light-adapted a-wave implicit time changed little with increasing flash intensity. The dark-adapted a-wave implicit time of the *rge* chicks also decreased with flash intensity at a similar rate, but was always about 5 mSec greater than that of controls. The light-adapted a-wave implicit time of the *rge* chicks decreased with flash intensity to a much greater extent than that of controls and was similar in magnitude and slope to that of the *rge* dark-adapted a-wave implicit time (I:R plot, Figure 6A, lower graphs). The b-wave I:R curve (Figure 6B, upper graphs) clearly showed the supernormal b-wave amplitude of the *rge* chicks in response to the brighter flashes. In the control chicks, the b-wave amplitude tended to plateau after 0 log cdS/m^2^ while that of the *rge* chicks continued to increase with increasing stimulus intensity, although it did flatten out at the highest intensities used. The b-wave implicit time of both dark- and light-adapted controls decreased with increasing stimulus intensity (Figure 6B, lower graphs). The mean dark-adapted b-wave implicit times of the *rge* chicks were similar to controls at the lower intensities when the b-wave amplitude was low, but were prolonged at the higher intensities as the b-wave amplitude increased (Figure 6B, lower left graph). The mean light-adapted b-wave implicit time of the *rge* birds was consistently longer than that of the controls across all stimulus intensities (Figure 6B, lower right graph).

### Comparison of a-wave leading edge

To facilitate the comparison of the slope of the leading edge of the a-wave, a comparison was made between control and affected a-waves by normalizing the a-wave amplitude of the affected chicks to that of the control chicks. Figure 5 shows dark- and light-adapted normalized a-wave responses averaged from eight control and eight affected chicks aged between 13 and 22 days old. The affected birds had a delayed onset of the a-wave down slope. Additionally, the initial stages of the down slope was not as steep as that of the control birds. There was also an increase in a-wave implicit time. The a-wave changes were similar between dark- and light-adapted conditions.

### Changes in maximal electroretinogram amplitudes with age

To investigate the duration of the supernormal b-wave with age and to chart the deterioration in ERG amplitudes with age in the *rge* birds, we plotted the mean maximal a-wave and mixed b-wave measured from the dark-adapted intensity series against age (Figure 4). The control birds showed a marked increase in maximal a-wave amplitude over the first few weeks of age, followed by a slower increase with age, over the ages investigated. *rge* chicks also showed an initial increase in maximal a-wave amplitude over the first week of age to reach maximal levels at seven days of age. Over the following week the maximal a-wave response decreased markedly and thereafter only slowly decreased with age. The control chicks showed a progressive increase in mean maximal b-wave amplitude with age, but not the marked increase over the first few weeks of age seen in the maximal a-wave response. The *rge* chicks had a marked increase in mean maximal b-wave amplitude over the first week of age. The resulting supernormal b-wave was maintained for a few weeks and then by 56 days of age the mean b-wave amplitude had decreased to be similar in amplitude to that of the controls. From that age onwards there was only a gradual decrease in maximal b-wave amplitude over the study period.

### Naka-Rushton fitting

**Figure 7:**
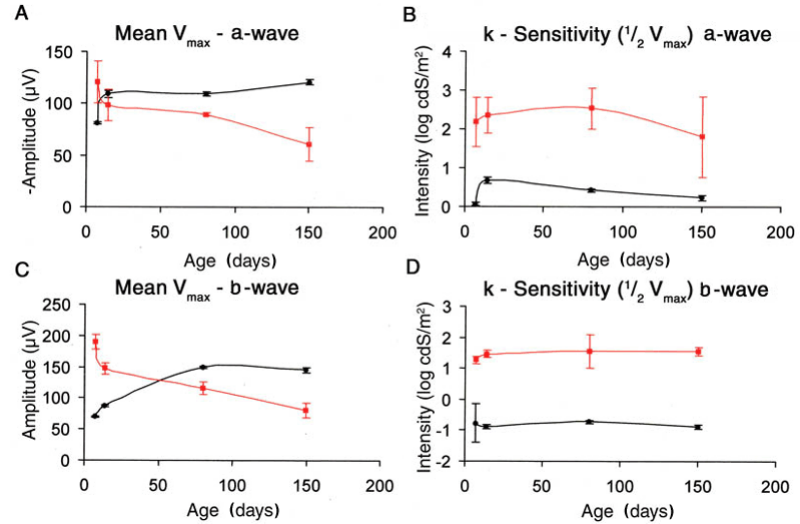
Graphs of mean a- and b-wave Vmax and sensitivity (k) plotted against age. Graphs showing the results for Vmax (**A** and C) and k (retinal sensitivity, **B** and **D**) derived from the Naka-Rushton fits of intensity-response curves. They are shown as mean (1x±SEM) for a-wave (**A** and **B**) and b-wave (**C** and **D**). The value for a-wave mean Vmax was greater in the 7-day-old *rge* birds but declined with age. The values for b-wave Vmax were initially greater in the affected compared to the control birds (7 and 28 days old) but decreased with age. The k value of the affected birds was significantly elevated (i.e., the retinas were less sensitive) compared to controls at all ages examined and remained so for the different ages tested. Note that for the a-wave amplitude the amplitudes were multiplied by -1 to make them positive.

Dark-adapted a- and b-wave intensity responses curves were fit to the Naka-Rushton formula. Figure 7 shows the results of mean Vmax and k (intensity at 1/2Vmax), which is considered a measure of retinal sensitivity. The value of k for both a- and b-waves of *rge* birds was significantly higher at all time points analyzed than that of normal controls. The value was approximately a factor of 1.5 to two log units higher, indicating that the *rge* birds had much reduced retinal sensitivity. The difference in sensitivity remained similar over the ages analyzed. The Vmax of both a- and b-waves of the *rge* chicks decreased with age. At seven days of age both a- and b-wave Vmax were higher in the *rge* chicks than in the normal controls. The Vmax of the *rge* chick b-wave in 7 and 14 day-old chickse reflected the supernormal b-wave as already described (see above and Figure 4B). The values for Vmax, particularly that of the b-wave, increased with age in the normal controls, whereas the value progressively decreased with age in the *rge* birds.

### Cone flicker responses

**Figure 8:**
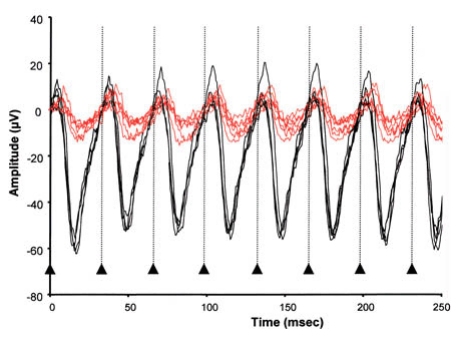
30 Hz flicker tracings from 1-day-old *rge* and control chicks. A comparison of 30 Hz flicker tracings of 1-day-old *rge* chicks (red lines) and control chicks (black lines) at a flash light intensity of 0.4 log cdS/m^2^ superimposed on a background light of 30 cd/m^2^. The amplitudes of the responses from the *rge* chicks are markedly lower than the controls (n=4 control and five *rge* chicks). Arrowheads with dotted lines indicate flash onset.

Cone flicker responses (30 Hz superimposed on a background light of 30 cd/m^2^) of the *rge* chicks were reduced in amplitude from one day of age (Figure 8) and continued to decrease with age (data not shown). The mean amplitude of the 30 Hz flicker ERG response in *rge* chicks of all ages investigated was 5.6±4.2 μV compared to 90.1±11.1 μV for controls (p<0.0001). There was a slight but not significant increase in mean implicit time compared to 1-day-old controls. By seven days of age the difference was significant (*rge* chicks 39.7±1.7 mSec; control chicks 36.8±1.7 mSec; p=0.0037). The implicit time further increased with age as the amplitude decreased.

### Retinal histology

**Figure 9:**
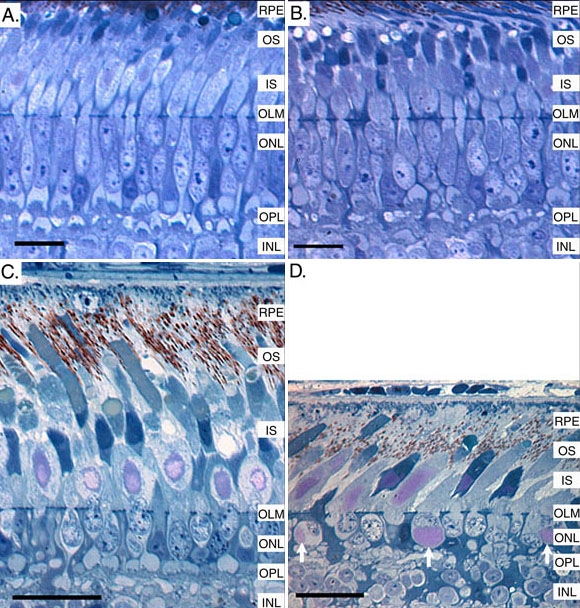
Semithin sections of the outer retina of control and *rge* birds at one and 270 days of age. Semithin retinal sections showing the outer retina of a control (**A**) and *rge* (**B**) chick at one day of age and a control (**C**) and *rge* (**D**) chicken at 270 days of age. The 1-day-old *rge* retina had only subtle morphological retinal abnormalities, involving slight dilation of inner segments and some disorganization of the outer plexiform layer. The affected birds had a slow thinning of the photoreceptor layer with age. By 270 days of age the *rge* bird had a marked thinning of the photoreceptor layer and further disruption of the outer plexiform layer. There was also the presence of displaced glycogen deposits adjacent to photoreceptor nuclei (arrows). Photomicrographs adapted with permission from: Montiani et al 2005 [15]. Size bars for **A** and **B** represent 10 μm. Size bars for **C** and **D** represent 20 μm. RPE indicates retinal pigment epithelium; OS indicates photoreceptor outer segments; IS indicates photoreceptor inner segments; OLM indicates outer limiting membrane; ONL indicates outer nuclear layer; OPL indicates outer plexiform layer; INL indicates inner nuclear layer.

Figure 9 shows representative histology of the outer retina of 1- and 270-day-old control and *rge* birds. Retinal thicknesses are comparable in 1-day-old chicks, and there were only subtle changes in the retina of the *rge* chick (Figure 9B). These include slight dilation of photoreceptor cell bodies and disorganization of the outer plexiform layer. Other retinal layers appeared normal. With increasing age there is a progressive thinning of the photoreceptor layer of the *rge* bird. Figure 9D shows a section from a representative 270-day-old *rge* bird demonstrating thinning of the photoreceptor layer compared to the age-matched control (Figure 9C). *rge* retinas at this age have misplaced glycogen deposits adjacent to photoreceptor nuclei within the outer nuclear layer, while control retinas have glycogen deposits within the inner segments as part of the hyperboloid or paraboloid. Thus, although the *rge* birds have structural abnormalities in the outer retina from an early age, they only develop a slowly progressive thinning of the photoreceptor layer with age.

## Discussion

The *rge* chick has an abnormal ERG from hatch. A- and b-wave thresholds are elevated for both dark-adapted and light-adapted responses, and the waveform is of an unusual shape. However the most striking feature of the *rge* ERG is a supernormal b-wave in response to brighter flashes. This develops between one and seven days of age and is maintained for the first four to six weeks of life. By 56 days of age the b-wave has decreased in amplitude such that the maximal b-wave is comparable to that of control birds. There is only a slow deterioration in ERG amplitudes with age after this time. It is interesting to note that during the age period that the supernormal b-wave is present the chicks undergo a marked deterioration in vision as assessed by an opticokinetic device [14].

ERG response thresholds of the *rge* birds were elevated compared to age-matched controls at all ages. The dark-adapted thresholds were more markedly elevated than the light-adapted thresholds such that there was little difference between the light- and dark-adapted criterion thresholds for both a- and b-waves. This, coupled with the shifting of the intensity response curves toward brighter intensities, suggests a marked reduction in rod-driven responses, or alternatively a marked reduction in rod sensitivity. Our previously published histopathological description of the *rge* defect showed that there was only a slow loss of rod photoreceptors. Mislocalization of rhodopsin immunoreactivity within rod photoreceptors cells was detectable from 13 days of age, suggesting that, although rods were present, there was an early abnormality that was having an effect on the rods. Although the apparent lack of rod responses may suggest similarities to congenital stationary night blindness (CSNB) the ERG changes differ from some forms of CSNB. For example, with X-linked and recessive forms of CSNB there is a lack of b-wave resulting in a negative ERG waveform [17]. This is clearly not the case with the *rge* chicken where supernormal b-waves are present, a feature that has not been described in CSNB patients to the authors' knowledge.

The relatively (compared to many diurnal species) small rod component in the normal chicken ERG in response to bright flashes is shown when the dark- and light-adapted I:R plots of control birds are examined. At the bright flash intensities the dark-adapted amplitudes are only slightly greater than the light-adapted responses. This would be anticipated because the normal chicken retina has a high proportion of cones compared to rods (the percentage of rods has been reported to vary from 14% centrally to 33% peripherally [4]) and is in contrast to many diurnal mammals, including humans, where rods account for somewhere in the region of 95% of the photoreceptors [18]. Species with a more rod-dominated retina have a cone-derived ERG of an amplitude lower than the rod-derived ERG.

As well as the apparent abnormalities in the rod driven ERG the *rge* birds also have abnormalities in the light-adapted ERGs. Light-adapted ERGs in control chickens are assumed to derive from cone pathway response, the background light was of an intensity that is recommended for the suppression of mammalian rod responses [19]. We make the assumption that this is true also for the chicken, and in control birds it certainly resulted in an elevation of response thresholds as would be expected. In the mutant chick this assumption may not hold true, because the phenotype may result in an elevation of rod response threshold such that the background light used would not completely suppress the rod-driven responses. A lack of rod sensitivity, meaning that rod function is not suppressed by the background light normally used to record light-adapted responses, has been described in other dystrophies, for example, the RPE65 knockout mouse [20].

Naka-Rushton fits of a- and b-wave responses with derivation of the k value (flash intensity that induces an amplitude of 1/2Vmax), which is considered a measure of retinal sensitivity, showed the *rge* birds have a marked reduction in retinal sensitivity. This is in keeping with the delayed threshold responses and the shifting of the intensity response curves to the right.

The leading slope of the a-wave of the *rge* chicks was delayed and more shallow than that of the normal controls. In primates, the initial portion of the a-wave is dominated by the photoreceptor response [21], although, particularly in the photopic response, the later portions of the a-wave were shaped by significant post-receptoral contributions [22]. If the initial portion of the chicken a-wave also represents primarily photoreceptor function then the delay in onset and more shallow slope of the leading edge of the a-wave as well as the raised threshold and shift of the light-adapted I:R curves to the right would, suggest there is photoreceptor dysfunction from an early age. Clearly there are abnormalities of the *rge* ERG that are likely to originate from alterations of the inner retinal components of the ERG. The b-wave normally has oscillatory potentials (OPs) superimposed on it. It is thought that the OPs originate primarily from inhibitory feedback loops from inner retinal neurons [23]. The lack of OPs in the *rge* chick ERG suggests that there could be an abnormality of inner retinal cells or the circuitry feeding into those cells. Immunohistochemistry showed that amacrine cell loss was not an early feature of the *rge* phenotype [15]. The supernormal b-wave also pointed to abnormalities affecting circuitry or input to circuitry of the inner retina. There are several reports in the literature of human patients having abnormally enhanced ERG waveforms [24-29]. The ERGs of these patients typically had b-waves that in response to bright flashes were supernormal in amplitude and had an increased implicit time. Weaker flashes resulted in ERGs with abnormally low amplitudes and markedly increased implicit times. A subset of patients with supernormal ERGs proved to have enhanced S-cone syndrome where there is a lack of rods and a preponderance of S-cones due to abnormalities in a transcription factor (NR2E3), important in determining the fate of photoreceptor progenitor cells [30,31]. Affected humans and NR2E3 knockout mice have increased numbers of S-cones and reduced rod function [30,32]. It seems unlikely that a similar defect underlies the *rge* dystrophy, because our previous histopathological study did not reveal any obvious differences in the relative numbers of different photoreceptor types. Unlike the situation in S-cone syndrome rod photoreceptors were present at retinal maturation in apparently normal numbers [15]. Furthermore, known transcription factors that govern photoreceptor progenitor fate (such as NR2E3 and Nrl [33]) do not map to the *rge* region of the chicken genome. There is an additional human dystrophy characterized by supernormal b-waves that was initially thought to be due to an abnormality in receptor cGMP activity [24]. Subsequent detailed ERG analysis of four patients failed to reveal abnormalities in outer segment transduction activation or deactivation and implicated the inner retina as the site of disease action [29]. The name "supernormal and delayed rod ERG syndrome" was suggested [29]. The *rge* chicken ERG shares some similarities with that of patients with this dystrophy, namely reduced responses to dimmer flashes and enhanced responses to brighter flashes. However, in the affected human patients it was the rod b-wave that was supernormal in response to bright flashes [29], whereas in the *rge* chicken it is likely the cone response that drives the enhanced b-waves. Although previously discussed, the possibility remains that a marked reduction in rod sensitivity could mean that the light-adapted ERG has a rod component in the *rge* chicken.

Following the development and then loss of the supernormal ERG b-wave there is a slowly progressive deterioration in ERG amplitudes. Histological studies show that there is also a slowly progressive thinning of the photoreceptor layer. The retinal thinning occurs as the globe of the affected birds increases in size (both in axial length and in radial globe diameter). The alteration in globe size appears to be secondary, because it is preceded by marked ERG abnormalities, deterioration in vision, and histological retinal abnormalities. Once the globe starts to enlarge, this is likely to contribute to the thinning of the retina that develops, and indeed, the birds can eventually develop changes associated with retinal stretch such as splits in Bruch's membrane (lacquer crack lesions) [34]. In humans there is an association between increased axial globe length and a diminution of a- and b-wave amplitudes [35]. This association does not appear to occur in chicks with form deprivation or defocus, although one study found a reduction in OPs [36] and another study found an increase in cone photoreceptor sensitivity [37]. In view of these previous studies in chicks, it seems more likely that the gradual reduction in ERG amplitudes with time are because of slow deterioration in retinal function due to the disease process rather than secondary to the globe enlargement that develops.

In summary, the *rge* chick has an unusual retinal dystrophy that results in a rapid loss of vision (as previously documented [14]) accompanied by abnormal ERG waveforms. The ERG of *rge* birds showed abnormalities of both rod and cone responses with elevated response thresholds. Inner retinal contributions to the ERG are also abnormal, with a lack of OPs and a supernormal b-wave in the earlier stages of the disease. In the *rge* chick, functional vision was lost at an early age, but there was only a slow, but progressive, loss of ERG waveforms. Additional studies are needed to further investigate the changes that underlie the abnormal ERG waveform of the *rge* chick. The use of a long flash ERG technique could be used to separate ON and OFF components to see if an alteration in one or both of these responses that are superimposed on each other in the regular short flash ERG is responsible for the altered waveform shape. A dissection of the *rge* ERG by administration of intravitreal drugs to block various neuron responses may help in the understanding of the origin of the changes underlying this unusual retinal dystrophy.
